# Immune Response to Sipuleucel-T in Prostate Cancer

**DOI:** 10.3390/cancers4040420

**Published:** 2012-04-18

**Authors:** Eddie Thara, Tanya B. Dorff, Monica Averia-Suboc, Michael Luther, Mary E. Reed, Jacek K. Pinski, David I. Quinn

**Affiliations:** Keck School of Medicine at USC, University of Southern California Norris Comprehensive Cancer Center, 1441 Eastlake Avenue, Suite 3440, Los Angeles, CA 90033, USA

**Keywords:** castrate resistant prostate cancer, immunotherapy, biomarkers, sipuleucel-T, immune response

## Abstract

Historically, chemotherapy has remained the most commonly utilized therapy in patients with metastatic cancers. In prostate cancer, chemotherapy has been reserved for patients whose metastatic disease becomes resistant to first line castration or androgen deprivation. While chemotherapy palliates, decreases serum prostate specific antigen and improves survival, it is associated with significant side effects and is only suitable for approximately 60% of patients with castrate-resistant prostate cancer. On that basis, exploration of other therapeutic options such as active secondary hormone therapy, bone targeted treatments and immunotherapy are important. Until recently, immunotherapy has had no role in the treatment of solid malignancies aside from renal cancer and melanoma. The FDA-approved autologous cellular immunotherapy sipuleucel-T has demonstrated efficacy in improving overall survival in patients with metastatic castrate-resistant prostate cancer in randomized clinical trials. The proposed mechanism of action is reliant on activating the patients’ own antigen presenting cells (APCs) to prostatic acid phosphatase (PAP) fused with granulocyte-macrophage colony stimulating factor (GM-CSF) and subsequent triggered T-cell response to PAP on the surface of prostate cancer cells in the patients body. Despite significant prolongation of survival in Phase III trials, the challenge to health care providers remains the dissociation between objective changes in serum PSA or on imaging studies after sipleucel-T and survival benefit. On that basis there is an unmet need for markers of outcome and a quest to identify immunologic or clinical surrogates to fill this role. This review focuses on the impact of sipuleucel-T on the immune system, the T and B cells, and their responses to relevant antigens and prostate cancer. Other therapeutic modalities such as chemotherapy, corticosteroids and GM-CSF and host factors can also affect immune response. The optimal timing for immunotherapy, patient selection and best sequencing with other prostate cancer therapies remain to be determined. A better understanding of immune response may help address these issues.

## 1. Introduction

Prostate cancer has become a major focus for translational and clinical immunotherapy research, due to the fact that it is common, affecting over 200,000 men in the United States each year [1], and has a relatively long natural history allowing a longitudinal assessment of the impact immune therapy. For advanced prostate cancer, though androgen deprivation therapy (ADT) is highly effective, the majority of patients will progress to castration-resistant cancer (CRPC) after two years of treatment [2]. This progression often intially manifests as rising PSA which then may be followed by disease progression and worsening clinical symptoms over several months to years. This ability to detect early disease progression makes immune therapy, which takes time to initiate anti-tumor response, more feasible than in other solid tumors. In addition, while docetaxel chemotherapy significantly prolongs survival for men with metastatic CRPC, the adverse side effects including neuropathy, neutropenia and generalized fatigue limit its use, especially in an elderly population with frequent bone marrow compromise related to the bony predilection of prostate cancer. These factors provide impetus for pursuit of alternative, non-chemotherapy approaches. With the approval of sipuleucel-T, immunotherapy now provides a viable option as an adjunctive to the hormonal treatment of metastatic CRPC. In this review we will outline the biologic underpinnings and early clinical results of sipuleucel-T, as well as other promising immune therapies in development for prostate cancer.

## 2. The Immune System and Cancer

The immune system arises from the hematopoietic stem cells of the bone marrow, producing two major lineages: myeloid and lymphoid progenitor cell lines. Myeloid cell lineage consists of monocytes, macrophages, dendritic cells, megakaryotcytes, and granulocytes. The lymphoid cell lineage includes T cells, B cells and Natural killer (NK) cells. These cell lineages ultimately produce the cellular components of the innate and adaptive immune systems. Possessing pattern recognition receptors which respond to general characteristics found on pathogens, the innate immune system include polymorphonuclear leukocytes (PML), monocyte/macrophages, eosinophils, platelets, NK cells, basophils, and mast cells. Antigen Presenting Cells (APCs), the dendritic cells (DC) and macrophages, function as a key link between the innate and adapative immunity. By expressing class II major histocompatibility cell surface molecules, APCs communicates with helper T cells, naive/memory B cells as well as NK cells, which then effectively activates the adaptive immune system, inducing differentiation of B cells to plasma- antibody producing cells and /or differentiation of T cells into cytotoxic T cells.

The intact immune system does not only defends our bodies against microbial infection, but also an active barrier to tumor formation and progression. Immune surveillance recognizes and eliminates the majority of nascent tumors and its failure has a prominent role in tumorigenesis. The deficiency in function of CD8+ cytotoxic T lymphocytes (CTLs), CD4+ Th1 helper T cells, or natural killer (NK) cells have been correlated with a significant increase in tumor incidence [3]. Compared to general population, patients with immunodeficiency due to HIV infection are at higher risk of developing several types of cancers, including lung, anal, liver, sarcoma and lymphoma [4]. Even though the incidence of AIDS-defined malignancies has decreased in highly-active-antiretroviral-therapy era, individuals diagnosed with AIDS at young age remain with elevated risk of certain cancers [5]. As such, the development of neoplasia in HIV-infected patients is similar to that observed in solid organ transplant recipients who receive chronic immunosuppressive agents, as well as in patients with profound cell-mediated immune deficiencies [4]. In the case when the immune system fails to stop a solid tumor formation, the antitumoral responses continue in the body. T cells, B cells, natural killer (NK) cells, DC and macrophages have the capacity to infiltrate solid tumors in humans and animals [6,7]. Immunohistochemistry studies have concluded that in the majority of solid tumors, the density of tumor-infiltrating leukocytes, like lymphocytes and DCs, inversely correlated with unfavorable features such as lymph node and distante metastasis and overall survival [8,9]. Patients with colon tumors infiltrated with CTls and NK cells had better prognosis than patients with tumors lacking such infiltration of killer lymphocytes (4). The extent of lymphocytes infiltration in prostate cancer tissue was also associated with improved prognosis [10]. Likewise, mice with immunodeficiencies in both T cells and NK cells are significantly more susceptible to tumor development than controls. On the other hand, tumors can also avoid eradication by evading immunological detection [11].

Tumors utilize several mechanisms to escape immune detection. These mechanisms include class I HLA downregulation which causes decreased susceptibility to CD8 CTL lysis [12], programmed death (PD)-1 ligand expression [13] and Fas-ligand expression, all of which induce apoptosis or de-activation of infiltrating lymphocytes [14]. Inhibitory cytokines, indoleamine 2,3-dioxygenase [15] and nitric oxide synthetase [16] secreted by tumor cells are also other approaches of evading immune eradication. Other mechanisms for aborogating the imune system by tumor cells include local production of cytokines such as vascular endothelial growth factor (VEGF), interleukins-6, 8 and 10 (IL-6, IL-8, IL-10), and tumor growth factor beta (TGF-beta), which create a tolerogenic phenotype in antigen presenting cells and allow tumor cells to avoid detection by various facets of the immune system. In that regard, inflammation is implicated in the pathogenesis and progression of cancer with paralleled effects on the immune system [17]. The balance between these factors shielding the tumor from the immune system as a cancer progresses, which might reversed as part of immunotherapuetics, and the hosts ability to develop an effective response are key. These issues and the spectra of mechanisms impacting the immune system in cancer have recently been reviewed [18,19].

## 3. Immune Therapy: Antigens and Activators

Selecting an appropriate antigen for anti-neoplastic immunotherapy is critically important; the antigen must be present on tumors from a wide range of people, should be expressed only on cancer cells and not normal host cells as much as possible, and must be capable of eliciting a strong immune response. Prostate cancer-associated antigens which meet these criteria and have been the target of therapeutic vaccines include PSA, prostate specific membrane antigen (PSMA) and prostatic acid phosphatase (PAP) [20,21]. An alternate approach is to utilize whole tumor cell vaccines, in order to present a broad array of tumor antigens to the immune system [22]. Table 1 summarizes immune agents which have undergone clinical investigation in advanced prostate cancer.

**Table 1:** Immune agents for prostate cancer discussed in this review, with their target(s) and vehicles.

Agent	Target(s)	Vehicle and Co-stimulants
PROSTVAC-VF [21]	Prostate Specific Antigen	FowlPox and Vaccinia viral vectors with B7.1, ICAM-1, and LFA-3 costimulants
GVAX [25,26,27]	Multiple	LNCaP and PC-3 cell lines transfected to secrete GM-CSF
Ipilimumab [28,29,30]	CTLA4	Antibody
Tremelimumab [31]	CTLA4	Antibody
MDX-1106 [32]	Programmed cell death-1 receptor (PD-1)	Antibody
Sipuleucel-T [33,34]	Prostatic acid phosphatase	Dendritic cells cultured ex-vivo with recombinant fusion protein of PAP and GM-CSF

Prostvac-VF is an example of a single antigen-targeted immunotherapy. It consists of a recombinant plasmid construct composed of human PSA with vaccine or fowl pox viruses which is subsequently combined with co stimulatory molecules (B7.1, ICAM-1, and LFA-3). Prostvac-VF serves as a tumor associated antigen, and thus effectively instigates a T cell-mediated response [23,24]. Kantoff and colleagues in a randomized, controlled, multicenter and blinded phaseII study treated eighty-two patients with Prostvac-VF while forty patients received the control vector. After three years, patients who were administered Prostvac-VF had an overall survival (OS) of 30% *versus* control group which had OS of 17%. The treatment group also had longer median survival by 8.5 months and a 44% reduction in death rate in men with minimally symptomatic castration-resistantmetastaticprostate cancer (mCRPC). Increased elispot reactivity to PSA was associated with better overall survival. Ongoing randomized clinical trials will more rigorously test the efficacy of Prostvac-VF, both independently and in combination with standard chemotherapy regimens [21].

GVAX is an example of a multi-antigen approach. It is derived from human prostate cancer cell lines (LNCap and PC-3) which are inactivated by radiation, and is coupled with granulocyte-macrophage colony-stimulating factor (GM-CSF) stimulation [35,36]. Adeno-associated viral vectors encoding the human GM-CSF gene under a viral promoter are initially transduced inside prostate cancer cell lines, which are then cultured and later irradiated to prevent proliferation after injection [37]. The product is administered via subcutaneous injection. While GVAX Phase I and Phase II clinic trials suggested efficacy [25,38], two Phase III trials were unfortunately unable to reproduce similar results. These trials, VITAL 1 and VITAL 2, aimed to compare GVAX in combination with and in sequence with docetaxel but were prematurely terminated due to imbalance in deaths in the two study arms [26,27]. The excess deaths in the experimental arm occurred despite balanced patient baseline characteristics [27].

A third category of immunotherapy is non-antigen targeted activation, such as releasing the cytotoxic T-lymphocyte antigen (CTLA)-4 mediated inhibion of cytotoxic T cell activation. Releasing CTLA-4 inhibition allows a net sustained T-cell mediated immune response to be activated, even though inhibition impacts both “helper” and “suppressor” T-cell subsets [39]. The potency of this approach is highlighted by the severe auto-immune side effects which have been reported using the anti-CTLA4 antibody ipilimumab [30]. Ipilimumab was recently approved for the first line treatment of malignant melanoma, and has undergone significant testing in prostate cancer as well. Attempting to focus on the otherwise non-specific immune activation, the phase II trial explored the use of a single dose of radiotherapy to a site of bone metastasis 24–48 h prior to the first ipilimumab infusion, to induce apoptosis and generate prostate cancer antigens for presentation by APCs. Ipilimumab is currently in phase III clinical trials, both before and after docetaxel therapy for patients with metastatic CRPC. Sequencing of immune therapy with chemotherapy is also an important question, which is being addressed in an ongoing randomized phase II clinical trial comparing four monthly doses of ipilimumab as a single agent *versus* ipilimumab in combination with a single dose of docetaxel in patients with CRPC. These studies are underway but are yet to be published [29,40].

Early phase work with the monoclonal antibody MDX-1106 targeting the programmed cell death-1 receptor (PD-1), which binds B7-H1 on T cells as well as possibly some tumor cells to de-activate the T cells in a pathway parallel to CTLA-4, is underway [32]. The presence of the B7-H1 in prostate cancer aggregates has provided a molecular rationale for these clinical trials in patients with prostate cancer [41]. Recent work implicates myeloid derived stem cells with macrophage phenotypes, NK cells and lymphocytes in the progression, metastasis and development of therapeutic resistance in a number of cancers including prostate cancer [42,43,44]. The potential to specifically target these cellular mediators may provide a rational for newer therapies.

There are several other cellular methods for inducing immunotherapeutic activity. Allogeneic stimulation of the immune system has traditionally been achieved with myeloablative therapy and bone marrow or stem cell transplantation from a living donor for hematological malignancies such as chronic myeloid and acute myelogenous leukemia [45]. In this context, there is a dual therapeutic goal of leukemia elimination and engraftment of the donor marrow with resultant graft *versus* leukemia effect adding to the therapy. This therapy has evolved for other malignacies with the aim of inducing a graft *versus* cancer effect without significant myeloablation. The nonmyeloablative stem cell transplant concept was explored in renal cell cancer with initially promising results from the NCI, however, long term cancer control was disappointing and toxicity related to graft *versus* host disease (GVHD) was significant [46,47]. In addition, follow-up trials failed to reproduce the original encouraging results with high rates of GVHD related toxicity and lack of measurable graft *versus* tumor effect [48,49]. Myeloablative therapy with autologous stem cell rescue has been used in some cases of castrate-resistant prostate cancer using either chemotherapy or radioisotopes with some success [50,51]. The aim of these regimens was to give high therapeutic doses of agents and rescue the bone marrow rather than produce an immunologically mediated anti-cancer effect. Newer strategies such as selected donor lymphocyte infusions are in development to reduce the toxicity of this approach while maintaining the immune effects of allostimulation and, if successful, may have application in prostate cancer.

Other approaches to simulate a T cell response, including immunostimulatory monoclonal antibodies (mAb), are under investigation. Monoclonal antibodies can mediate antitumor effect by activating host immune function or by targeting tumors with conjugated cytotoxins or radioactivity. The fact that prostate cancer metastases often involve bone and lymph nodes, locations that receive high levels of circulating antibody and have been responsive to mAb therapies in other tumor types such as lymphoma, makes this approach an attractive therapeutic strategy. HuJ591 is an example of mAb to the prostate specific membrane antigen (PSMA) extracellular domain that is being explored in clinical trials [52]. Since prostate cancer is radiation sensitive, radiolabeled and unlabeled versions of HuJ591 have been evaluated for clinical efficacy in a phase II trial of 177-Lutetium (177Lu) linked to HuJ591 in 14 men with progressive castrate-resistant prostate cancer. The trial reported a PSA decline of >50 percent in one patient and four patients had disease stabilization [53]. Another phase I study of 111-Indium-labeled HuJ591 also demonstrated targeting of known metastatic sites in patients with other solid tumors, implying the drug’s potential as a vascular targeting agent [54]. Another PSMA-targeted monoclonal antibody, MLN2704, is conjugated to the maytansinoid antimicrotubule agent DM1 and delivers it directly to prostate tumor cells. A phase I/II trial tested MLN2704 in 61 men with progressive castrate-resistant prostate cancer [55]. While the antitumor activity was greatest at 330 mg/m^2^ every 2 weeks, including major PSA declines in four of six patients, the frequency of grade 2 and 3 peripheral neuropathy necessitated delay in treatment in most patients and further evaluation with different schedule will be required.

## 4. Sipuleucel-T

Sipuleucel-T (Provenge^®^, Dendreon) is a targeted autologous cellular immunotherapy for prostate cancer. The product consists of autologous dendritic cells which are activated *ex vivo* by a recombinant fusion protein comprised of prostatic acid phosphatase (PAP) and GM-CSF. PAP was chosen as a target due to its ubiquity in prostate cancer, with over 90 percent of prostate tumors expressing PAP, as well as ability to induce both humoral and cellular immune activation targeted towards prostate cancer cells. GM-CSF was incorporated due to its ability to potentiate the development and simulation of dendritic cells which can evoke an anti-tumor effect [56,57]. The cornerstone of sipuleucel-T is a recombinant DNA fusion protein (PA2024) comprised of GM-CSF and PAP. Treatment with sipulecel-T requires three main steps: CD54+ dendritic cells are first isolated from each individual patient via seven litre leukopheresis. These extracted cells are then incubated with the PA2024 fusion protein, *ex vivo* in a processing facility. Lastly, the activated autologous dendritic cells are infused into the patient. This process is repeated three times, at approximately 2-week intervals [58].

Phase I and II trials ascribed to the safety of sipuleucel-T as well as its ability to elicit an antigen-specific T-cell repsponse [58,59,60,61]. The drug gained approval by FDA after three randomized phase III clinical trials that looked at progression free survival and overall survival in patients who received the drug compared to placebo. The first two studies D9901 and D9902A were initiated in 1999 and 2001, respectively [62,63]. Both trials enrolled asymptomatic men with metastatic CRPC and blindly assigned them in 2:1 ratio to receive three infusions of sipuleucel-T or placebo. The placebo group did undergo leukopheresis, but their reinfusion consisted of one-third of their leukaphoresed cells which had not been incubated with recombinant fusion protein PA2024. The trial D9901 demonstrated no delay in the median time to disease progression (TTP), but rather significant improvement of overall survival from 21.4 months for the placebo group to 25.9 months for those treated with sipuleucel-T. The results of D9902A trial indicated similar median TTP between the two cohorts (10.9 weeks for sipuleucel-T *versus* 9.9 weeks for placebo, *p* = 0.719), but an increase in survival for patients treated with sipuleucel-T (*p* = 0.023) was associated with many important prognostic factors such as serum lactate dehydrogenase (LDH), PSA level, distribution and volume of disease in multivariate analysis.

In 2010, maturation of data from the IMPACT trial (9902B trial) confirmed the overall survival efficacy of sipuleucel-T [33]. Primary and secondary endpoints were overall survival and secondary endpoint of time to radiographic disease progression, respectively, in 512 patients with asymptomatic or minimally symptomatic metastatic CRPC, good performance status, serum PSA levels of ≥5.0 ng/mL and no visceral metastases. Meeting the primary endpoint, patients treated with sipuleucel-T experienced an extended median overall survival by 4.1 months compared to the placebo group (25.8 compared to 21.7 months) with a hazard ratio of 0.775 for risk of death (95% CIs 0.614–0.979; *p* = 0.032). Consistent with prior trials, the time to radiographic disease progression were similar in both arms (3.7 months and 3.6 months, *p* = 0.63).

Integrating the data from all three randomized trials, the risk of death from any cause is significantly reduced by 26.5% by sipuleucel-T (HR: 0.735; *p* < 0.001) and median overall survival is extended by 3.9 months [33,63,64]. Sipuleucel-T toxicity was generally limited to infusion reactions of limited, low-grade fever and chills, with resolution occuring by 48 h. Headache, influenza-like illness, hypertension and hyperhidrosis were other common side effects. The combined analysis comparing the complications in 601 patients receiving sipuleucel-T compared to 303 patients receiving a placebo indicates similar serious side effects (grade 3 or more by NCI criteria) in the sipuleucel-T treatment group (24.0%) compared to the placebo group (25.1%) [64]. Sipuleucel-T was approved by the United States Food and Drug Administration in April 2010.

## 5. Immune Response to Sipuleucel-T

None of the sipuleucel-T trials has demonstrated significant decline in PSA value or delay in time to disease progression in patients treated with sipuleucel-T, making it difficult for physicians to know if a patient has gained clinical benefit. It is increasingly evident that responses to immunotherapies are slower compared to androgen deprivation therapy, radiation and chemotherapy [65,66,67,68]. Cancer may remain stable or even progress for some months before protective immune responses become apparent. Counter intuitively, the initial vaccine-induced inflammatory response may be mistaken for tumor growth in much the same way as inflammation in the brain after radiation for glioma can produce pseudo-progression. In a similar fashion, disconcordant early rise in serum PSA and/or flare effect on bone scan in prostate cancer patients with osseous metastases is common with response to androgen pathway modulation and chemotherapy [69,70]. Revised endpoints for cancer vaccine trials that place greater emphasis on overall survival or long-term disease stability rather than time to progression have been proposed. The therapeutic intent is to minimize premature discontinuation of therapy and involves continuance of treatment despite minor progression [65,66,67,68].

Regardless of lack of an effect on PSA or other measurable disease parameters, humoral and cytotoxic-T cell responses were evident in preclinial and clinical studies and underscore the effect of sipleucel-T. In preclinical studies, treating rats with three doses of APCs incubated with a recombinant fusion protein consisting of rat PAP and rat GM-CSF at 14-day intervals elicited lymphocytic infiltrates in their prostate tissues [71]. Furthermore, removal of any component of the target antigen, APCs or GM-CSF resulted in attenuated treatment responses, implying the requirement of all three in prostate tissue immunomodulation.

In the earliest open-label non-randomized trial, Sipuleucel-T was administered to 13 patients with metastatic CRPC by two intravenous infusions, followed by three monthly subcutaneous injections of PA2024 at dosages of 0.3, 0.6 or 1.0 per injection [59]. T cell and B cell responses were evaluable using proliferation assays and ELISA, respectively. The trial demonstrated that little or no pre-existing T-cell proliferation occurred to PA2024 (PAP/GM-CSF fusion molecule) incubation, but all patients had T-cell proliferation response after infusion of sipuleucel-T, even after only one infusion. After sipuleucel-T treatment, 38% (10 of 26) of patients developed a T-cell response to PAP while 70% (19 of 27) of patients developed proliferation to GM-CSF. The specificity of T-cell stimulation by sipuleucel-T was shown by the absence of T-cell proliferation to recall antigen influenza and the naïve antigen KLH, as internal positive controls. In addition to T-cell activity, B-cell response can also be observed. Using specific ELISA on serum samples at baseline and every four weeks, 52% and 47% of patients developed antibodies to PAP and the PAP-GM-CSF fusion molecule after Sipuleucel-T therapy, respectively.

A second phase I clinical trial utilized three infusions of sipuleucel-T in men with CRPC who had progressed after chemotherapy, with a fourth dose given at week 24 if patients had stable disease [61]. The dose of sipuleucel-T was escalated from 0.2 × 10^9^ to 2.0 × 10^9^ nucleated cells/m^2^, with six patients treated with sipuleucel-T at the maximum dosage. All patients treated with sipuleucel-T developed T-cell proliferation response with documented minimal pre-treatment T cell proliferation response to PA2024. In addition, the 20 patients who developed the strongest immune response to PAP, by either T-cell proliferation assay or antibody titers, had a longer median time to disease progression compared to patient who did not mount the same degree of immune response (34 *versus* 13 weeks, *p* < 0.027) [61].

Demonstrated further by the immunologic assessment of the patients from the IMPACT trial, immune response was assessed in a subset of 134 patients and when present was associated with increased overall survival. Antibody titers against the PA2024 antigen were significantly more frequently observed at any post-baseline time point in those treated with sipuleucel-T (66.2 *versus* 2.9%), as were antibodies against PAP phosphatase (29 *versus* 1.4 percent) [33]. A pre-specified analysis showed that antibody responses to the antigens were associated with significant survival benefit (*p* < 0.0001 for PA2024 and *p*—0.008 for PAP). T-cell proliferation responses to PA2024 and PAP were more frequent in those treated with sipuleucel-T (73 *versus* 12 and 27 *versus* 8%, respectively), they did not reach significance relative to overall survival. In further analysis from the IMPACT trial, Stewart *et al*. reported that larger incremental increase in three cell-product parameters: total nucleated cell (TNC) count, CD54 up-regulation and CD54+ cell count; were associated with overall survival [72]. Assessing CD54 up regulation as part of quality control for processing the sipuleucel-T product is a surrogate of APC activation. Increase was observed in all products from the IMPACT trial, with greater magnitude of activation in the second and third products [73,74]. An additional method for assessing APC and T-cell activation from sipuleucel-T is measurement of their associated cytokine productions [75,76]. When the isolated peripheral blood mononuclear cells (PBMCs) from each isolated leukapheresis were incubated with PA2024 or GM-CSF alone, substantial increases in APC-associated cytokines levels such as IL-1α, IL-1β, IL-12p70, and TNFα, and T cell activation-associated cytokines like IL-2, IL-4, IL-5, IL-10, IL-17, IFNγ, and TNFα were noted with PA2024, but not with GM-CSF. Additionally, the CD4+ and CD8+ T cells generated by culture with PA2024 also expressed a signature of enhanced activation markers when assessed by flow cytometry (expression of CD134, CD137, CD278 and CD279). The increase cytokine production and T-cell activation were not observed when the cells were incubated with GM-CSF, suggesting that the effects were driven by PA2024 [76,77]. Using interferon gamma enzyme-linked immunospot (IFNγ ELISPOT), the pre-culture cells from the second and third products demonstrated a progressively increased antigen-specific T cell proliferation and memory response [73,78]. The pattern of activation may support the concept that the first infusion primes the immune system and subsequent infusions boost the response [73]. These data support the conclusion that broad engagement of the immune system contributes to the sipuleucel-T survival advantage seen in IMPACT. Table 2 summarizes the immune response data.

**Table 2:** Immune response and monitoring data.

Marker	Measuring Method	Correlation	References
T cell proliferation assay, T-cell surface protein expression	Radioactivity incorporated into proliferating cells, flow cytometry, IFNγ ELISPOT	T-cell activation and response to PA2024, targeting GM-CSF element and PAP	Small *et al*. [59] Burch *et al*. [61] Wesley *et al*. [76] Butterfield *et al*. [78]
B-cell Response (Antibody)	ELISA, Western blot	Antibody responses to PA2024 antigen, PAP and GM-CSF	Small *et al*. [59] Burch *et al*. [61] Kantoff *et al*. [33]
CD54+ cell count, CD54 up regulation, and TNC	Flow cytometry assay, allogeneic mixed lymphocytic reaction assay (allo-MLR)	APC activation	Stewart *et al*. [72]Sheikh *et al*. [74]
APC- and T-cell-associated cytokines	Conventional ELISA	APC and T-cell activation	Sheikh *et al*. [75]Wesley *et al*. [76]

## 6. Other Factors That May Affect Active Immunity and Immune Response

While sipuleucel-T has demonstrated efficacy and deploys immune responses by various measurements, a plethora of factors, such as other conventional concurrent therapies and host factors, may increase or diminish the host immune effects toward the prostate cancer. Studies have shown that augmentation of active cellular defense is not limited to immune-base therapy and could result to a degree from standard treatments for prostate cancer including hormone therapy and radiation. Interestingly, conventional androgen deprivation therapy produces profuse infiltration by activated and oligoclonal-specific T cells into prostate tumors and tissues [79]. In both animal and human models, androgen deprivation can bolster host lymphocyte levels and facilitate T-cell antigen-specific activation [80,81]. Furthermore, immune alterations induced by androgen deprivation may boost efficacies of immunotherapy. In a tumor-free mice model, administration of TCR- and CD28-mediated co-simulation and Ag-specific activation along with androgen deprivation led to more vigorously increased levels of peripheral T cells and their proliferations in lymphoid tissues [82]. More importantly, androgen deprivation accelerated normalization of the mice T- and B-cell levels following chemotherapy-induced lymphocyte depletion. By the same token, ADT in humans can also induce effector-cell response to stimulation, and the generation of a prostate tissue-associated IgG antibody response [83]. Radiation therapy activates of CD8+ cytotoxic response via induction of a wide spectrum of inflammatory cytokine production, including MHC molecules, B7 and other co-stimulatory molecules, adhesion molecules, death receptors and heat shock proteins in tumor cells, stroma, and vascular endothelium [84,85,86]. Using Western blot assays for protein expression, Nesslinger and colleagues reported that patients undergoing neoadjuvant hormone therapy, external beam radiation therapy and brachytherapy exhibited treatment-associated autoantibody generation as opposed to control patients treated with radical prostatectomy [87]. As such, several lines of evidence suggest that androgen deprivation and radiation therapy may potentiate the effect of immunotherapy and further study of this area is certainly warranted.

In contrast to androgen deprivation and radiation other concurrent therapeutic modalities may interfere with the cellular immune response. Glucocorticoid administration has many effects upon innate and acquired immunity. These include decreased production of pro-inflammatory cytokines [88] and inhibition of phagocyte function and migration [89,90]. However, the precise threshold of steroid exposure and duration of administration needed to suppress the immune system in an otherwise healthy person is still the subject of debate. Cytotoxic chemotherapy for prostate cancer routinely produces myelosuppression, and neutropenia and potentially fatal infections are potential uncommon side effects [91]. In contrast to the effects of cytotoxic agents on marrow suppression, chemotherapy can actually stimulate cellular cytotoxicity in similar manner to radiation. Chemotherapy drugs can cause tumor cell apoptosis leading to the release of tumor-associated antigens (TAAs), which may be taken up and presented by APCs. Moreover, particular chemotherapy agents can preferentially modulate regulatory T cells, up-regulate expression of TAAs, and decrease production of inhibitory tumor secreted cytokines [92,93,94]. In the preclinical setting, Garnett and colleagues noted that when docetaxel was given with a vaccine formulated from vaccinia virus encoding T-cell co-stimulatory molecule (TRICOM) which target CEA, immune response to stimulation was better than either agent alone [95]. Interestingly, docetaxel did not dampen the function of regulatory T-cells. In another preclinical mouse fibrosarcoma model, Garrido and coworkers demonstrated eradication of metastases when either docetaxel or autologous irradiated cells were added to protein bound polysaccharide K (PSK) but not by docetaxel alone, where the response was dependent on whether the tumor cells had irreversible or reversible MHC-1 alterations [96]. Hard and soft alterations in a variety of human leukocyte antigens (HLA) of the class 1 type are associated with response to immunotherapy: analysis in 2 melanoma patients treated with autologous vaccine, BCG and interferon-α demonstrated that regressing metastases expressed high levels of HLA-ABC molecules, while progressing lesions had low/intermediate levels of HLA class I and harbored structural defects (hard lesions) in MHC-I or β2-microglobulin genes. These molecule profiles may provide a means to response prediction as well as being a therapeutic target [97,98,99]. Another phase II study comparing pox viral-PSA vaccine given alone and in combination with low-dose docetaxel in men with mCRPC [100]. Again, docetaxel did not inhibit T cell-specific responses and the vaccine-docetaxel combination group had a longer progression-free survival (6.1 *versus* 3.7 months) compared to chemotherapy alone. Other studies with similar design also showed that patients who are treated with a vaccine first do better than those receiving chemotherapy alone [101,102]. Analyzing the data of 51 patients with mCRPC who were treated with sipuleucel-T or placebo, Petrylak reported that the overall survival was significant longer in patients who received the vaccine and then chemotherapy than those who received placebo and the same chemotherapy (34.5 *versus* 25.4 months, *p* = 0.023) [103]. Further study is ongoing to identify the best potential sequence of these therapeutic modalities. In the clinical practice setting where sipuleucel-T is being used, cellular proliferation such as CD54 and nucleated cell count is used as part of the manufacturing process with a several fold escalation is required for the product to be released back to the clinic for infusion. Failure to generate a cellular response during processing is unusual but has been observed in patients receiving corticosteroids, cytotoxic chemotherapy and GM-CSF in the weeks and months prior to initiating the sipuleucel-T process. In an analysis of cellular parameters in different patient groups treated with sipuleucel-T in clinical trials, Sheikh *et al*. reported a larger CD54 and T cell response in patients given therapy in the early neoadjuvant setting compared with more advanced disease castrate-resistant disease [104]. This suggests that early exposure to sipuleucel-T may be more efficient.

One area in which research is required relates to the patient’s inherited propensity to mount an immune response. Many immunological interactions are limited by HLA class I expression on tumor and immune cells. HLA classes are inherited in a Mendelian fashion. On that basis, HLA typing of individual patients has the potential to predict response and is a standard in many clinical trials that incorporate peptide vaccines [30,105,106]. HLA subsets appear to have minimal impact on response to CTLA4 antibody directed therapy in melanoma [107]. HLA genotype and whether prostate cancer cell expression of either PAP or GM-CSF is MHC dependent is an important and potential fertile area for future research.

## 7. Conclusions

Immune therapies are capable of changing the natural history of solid tumor including prostate cancer. Although the lack of PSA response or delay in time to disease progression make it difficult to assess which patients derive benefit from sipuleucel-T, the repeatedly observed survival benefit is noteworthy. In that regard, immunotherapies have begun to challenge our abilities to measure cancer response to treatment wit the conventional WHO or RECIST criteria. Work with ipilimumab has demonstrated that a variety of response can be associated with an improved outcome: (a) shrinkage in baseline lesions, without new lesions; (b) durable stable disease (in some patients followed by a slow, steady decline in total tumor burden); (c) response after an increase in total tumor burden; and (d) response in the presence of new lesions [66]. Each of these responses can overlap a variety of responses seen in measurable disease as per RECIST crieteria. Generally, the newly developed “Immune Response-related Criteria” call for leaving the patient on therapy despite the development of small new lesions or minor growth in established lesions to allow the patient to respond in other lesions or across the entire volume of disease after the typical window for response has passed. It is important to have early criteria for benefit subsequent benefit because the effects of many immunotherapies including sipuleucel-T are only evident starting a year after treatment. A example of this is the trend to reduced likelihood of bone pain at one year in patients given sipuleucel-T [108].

Evidence of frequent and significant humoral and cytotoxic-T cell responses to sipuleucel-T emphasize its mechanism of action, though a marker for benefit has unfortunately not yet been elucidated. Although sipuleucel-T has been primarily studied and approved in patients with metastatic disease, immunotherapies might be more efficacious if used earlier in the disease process due to better immune response in the host with less disease burden.

Future studies will focus on advancing sipuleucel-T to earlier stages of disease, where immune therapy has traditionally been felt to hold the greatest promise [34]. A recently publsihed study by Beer *et al*. compared patients with rising PSA and no evidence of metastases after prior surgery or radiation for localized prostate cancer in a phase II study where three months of LHRH agonist therapy was used in the control arm and sipleucel-T added in the experimental arm [34]. When these patients were followed beyond serum testosterone recovery, the PSA doubling-time in the sipleucel-T group was significantly higher than in the group given no sipleucel-T (median 5.1 *versus* 3.5 months, *p* = 0.038). This suggests an effect tumor cell kinetics with immunotherapy. In addition, patients in this study had evidence of ongoing immunity to PAP and PAP-GM-CSF move than 5 years after sipleucel-T therapy. The optimal sequence and combination of sipuleucel-T in prostate cancer remains to be determined. Ongoing studies are summarized in Table 3. Key questions also remain about the anitigens targeted in the sipuleucel vehicle and about the utility of other cell based immunotherapies in prostate cancer.

**Table 3:** Ongoing and upcoming clinical trials with Sipuleucel-T, derived from postings on the www.clinicaltrials.gov website.

Study Title	Study Design	Study Population	Primary Outcome	Secondary Outcomes	Status
An Open-Label Multicenter Study of Sipuleucel-T in Metastatic Castrate Resistant Prostate Cancer Patients Previously Treated With Sipuleucel-T on Dendreon Study P-11 (NCT00779402)	Open-label, uncontrolled, Phase II, multicenter study	Androgen dependent biochemical recurrence, previously treated on the P-11 study.	Immune responses	Safety Correlation between immune responses and survival.	Not yet recruiting
A Randomized, Open-Label, Phase 2 Trial Examining the Sequencing of Sipuleucel-T and Androgen Deprivation Therapy in Men With Non-metastatic Prostate Cancer and a Rising Serum Prostate Specific Antigen After Primary Therapy (NCT01431391)	Randomized, Open-Label, Phase 2 Trial	Androgen dependent biochemical recurrence.	Immune responses	Safety Immune responses PSA kinetics	Currently recruiting
An Open Label Study of Sipuleucel-T in Men With Metastatic Castrate Resistant Prostate Cancer (NCT00901342)	Multicenter, Open Label, Phase II Study	Patients who progressed on the IMPACT study who were on the control arm	Immune responses	Safety	Ongoing, but not currently recruiting
A Registry of Sipuleucel-T Therapy in Men With Advanced Prostate Cancer (NCT01306890)	Observational, prospective cohort	Metastatic, castration-resistant; population specified in product label.	Safety	Survival	Currently recruiting participants
A Pilot Study to Test the Feasibility and Immunologic Impact of Sipuleucel-T (Sipuleucel-T) Administered With or Without Anti-PD-1 mAb (CT-011) and Low Dose Cyclophosphamide in Men With Advanced Castrate-Resistant Prostate Cancer (NCT01420965)	Randomized, Open-Label, Phase 2 Trial	Metastatic, castration-resistant prostate cancer.	Safety Immune responses	Progression free survival and Overall Survival	Not yet recruiting
An Open Label, Phase 2 Trial of Immunotherapy With Sipuleucel-T (Sipuleucel-T^®^) as Neoadjuvant Treatment in Men With Localized Prostate Cancer (NCT00715104)	Single Center, Open Label, Phase II trial.	Localized disease, planning to undergo radical prostatectomy for definitive management.	CD3+ cell infiltration within prostate tissue	Serum immune responses Safety	Ongoing, but not recruiting patients
To Evaluate Sipuleucel-T Manufactured With Different Concentrations of PA2024 Antigen (NCT00715078)	Randomized, multicenter, single blind, Phase 2 trial	Subjects will receive sipuleucel-T, manufactured with 1 of 3 different concentrations of PA2024 antigen.	Cumulative CD54 up-regulation ratio between cohorts	Immune responses Survival	Ongoing, but not recruiting patients
